# Gastric neuroendocrine tumor arising from heterotopic pancreas

**DOI:** 10.1007/s12328-017-0795-3

**Published:** 2017-12-07

**Authors:** Takehiro Tanaka, Rika Omote, Noriko Okazaki, Hiroyuki Yanai, Tadashi Yoshino

**Affiliations:** 1 Department of Pathology, Okayama University Hospital, Okayama, Japan; 2 Department of Pathology, Okayama University Graduate School of Medicine, Dentistry and Pharmaceutical Sciences, Okayama, Japan

**Keywords:** Neuroendocrine tumor, Heterotopic pancreas, Stomach

## Abstract

There are few English reports on secondary tumors from heterotopic pancreas. Here, we describe a case of gastric neuroendocrine tumor (NET) arising from heterotopic pancreas. A 72-year-old woman underwent esophagogastroduodenoscopy as part of a general health check-up. An endoscopic examination revealed a submucosal tumor on the greater curvature of the gastric body. Laparoscopic and endoscopic cooperative surgery was performed. Histological diagnosis concluded that it was a Grade 1 NET arising from heterotopic pancreas. We report this extremely rare case of a NET presenting as a submucosal tumor, considered to have originated from heterotopic pancreatic tissue.

## Introduction

Neuroendocrine neoplasms of the stomach are relatively uncommon. Most neuroendocrine neoplasms are neuroendocrine tumors (NETs) that are well-differentiated, non-functioning enterochromaffin-like cell tumors arising predominantly in the corpus–fundus region. They encompass three distinct types—type I tumors are associated with autoimmune gastritis, type II tumors are associated with multiple endocrine neoplasia type 1, and type III are sporadic tumors [1]. Most Japanese NETs are type I.

Heterotopic pancreas is the most common form of heterotopia diagnosed in the stomach. Although its pathogenesis is controversial, it may be secondary to the abnormal location of developing pancreatic buds during embryogenesis. Heterotopic pancreas is commonly situated in the submucosa of the distal stomach, most often within 5 cm of the pylorus. It is seen during endoscopy as a solitary, umbilicated submucosal lesion with occasional erosion of the overlying mucosa. Heterotopic pancreas is composed of pancreatic acini, ducts, or islets. Ectopic pancreatic tissue can undergo secondary changes such as acute pancreatitis [2, 3], pseudocyst formation [4], development of mucinous cysts [5], and pancreatic carcinoma [6–8]. Neoplasms arising from ectopic pancreas are extremely rare. There has been only one previous report of NET occurring from ectopic pancreas [9]. Here, we report an extremely rare case of a NET presenting as a submucosal tumor, considered to have originated from ectopic pancreatic tissue.

## Case report

**Fig. 1:**
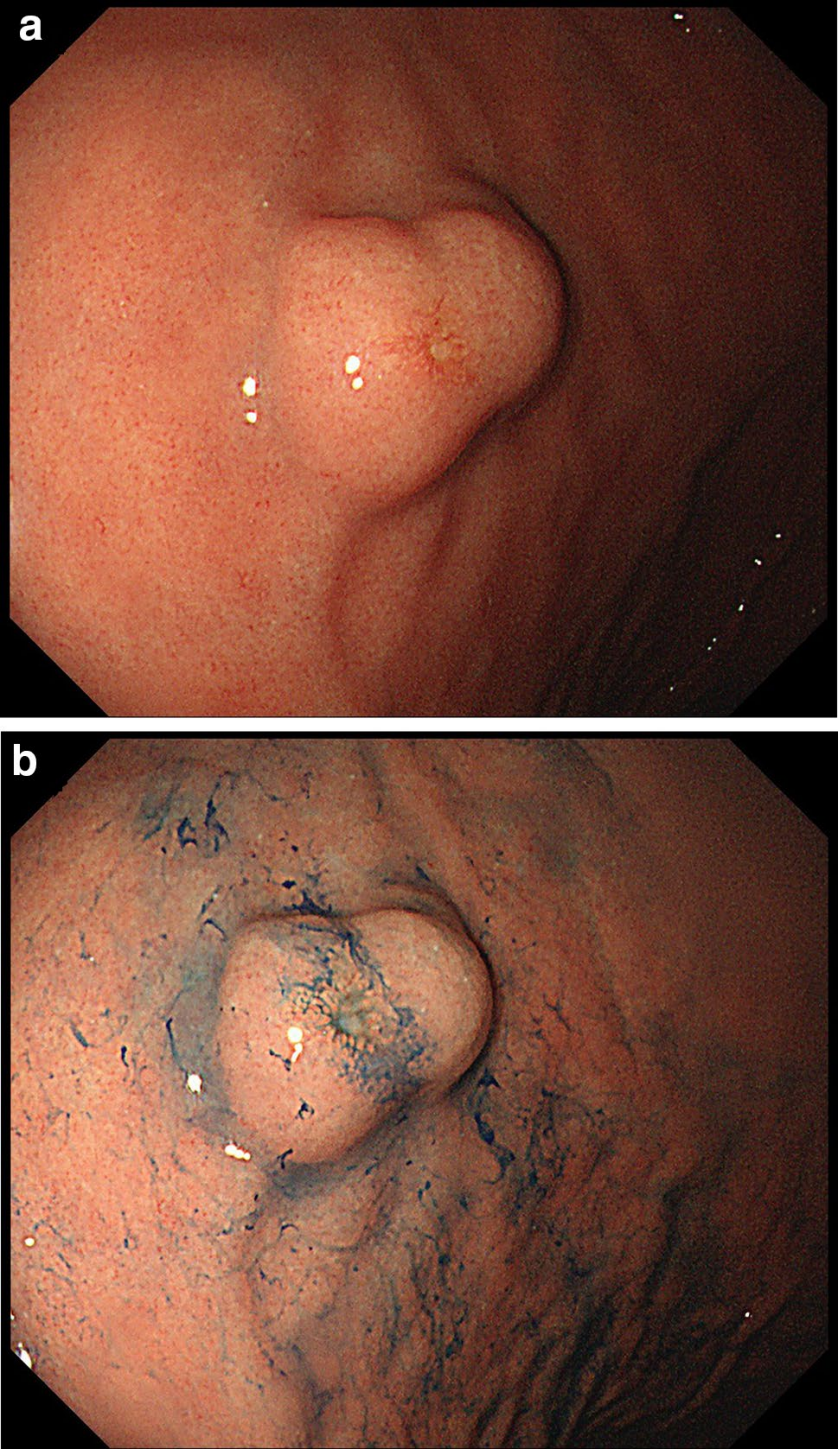
Endoscopic appearance of an elevated lesion on the body of the stomach. **a** The tumor had a smooth surface with a central depression and a small erosion on top. **b** Indigo carmine staining of the tumor

**Fig. 2:**
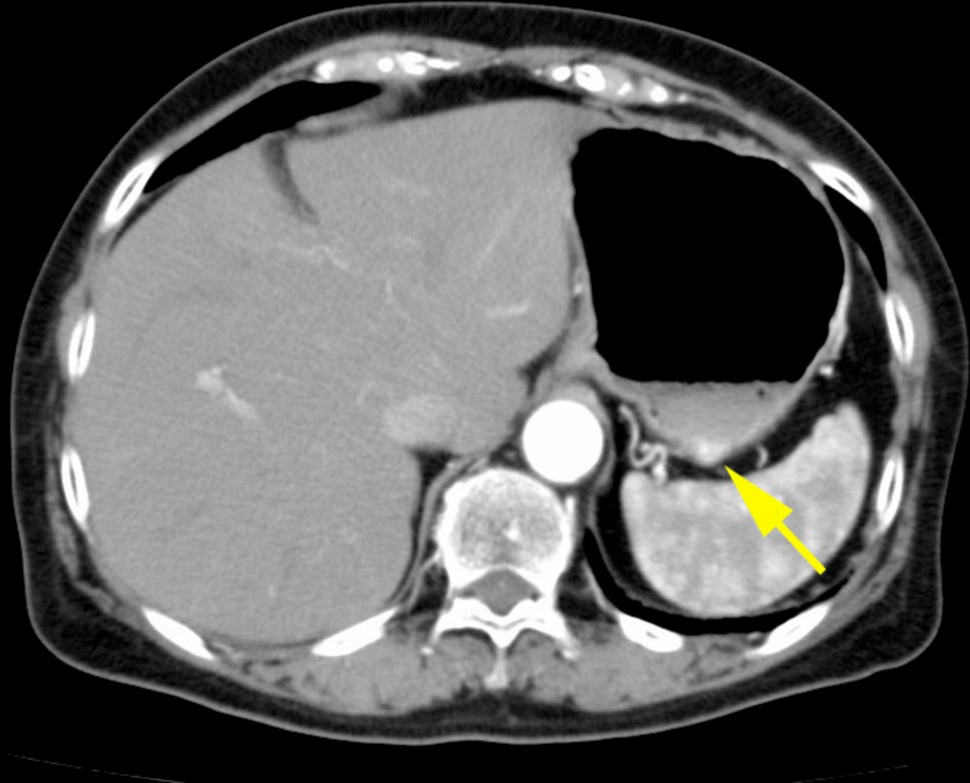
Contrast-enhanced computed tomography imaging. A low-attenuated tumor on the greater curvature of the stomach, measuring 16-mm diameter, with slow enhancement

**Fig. 3:**
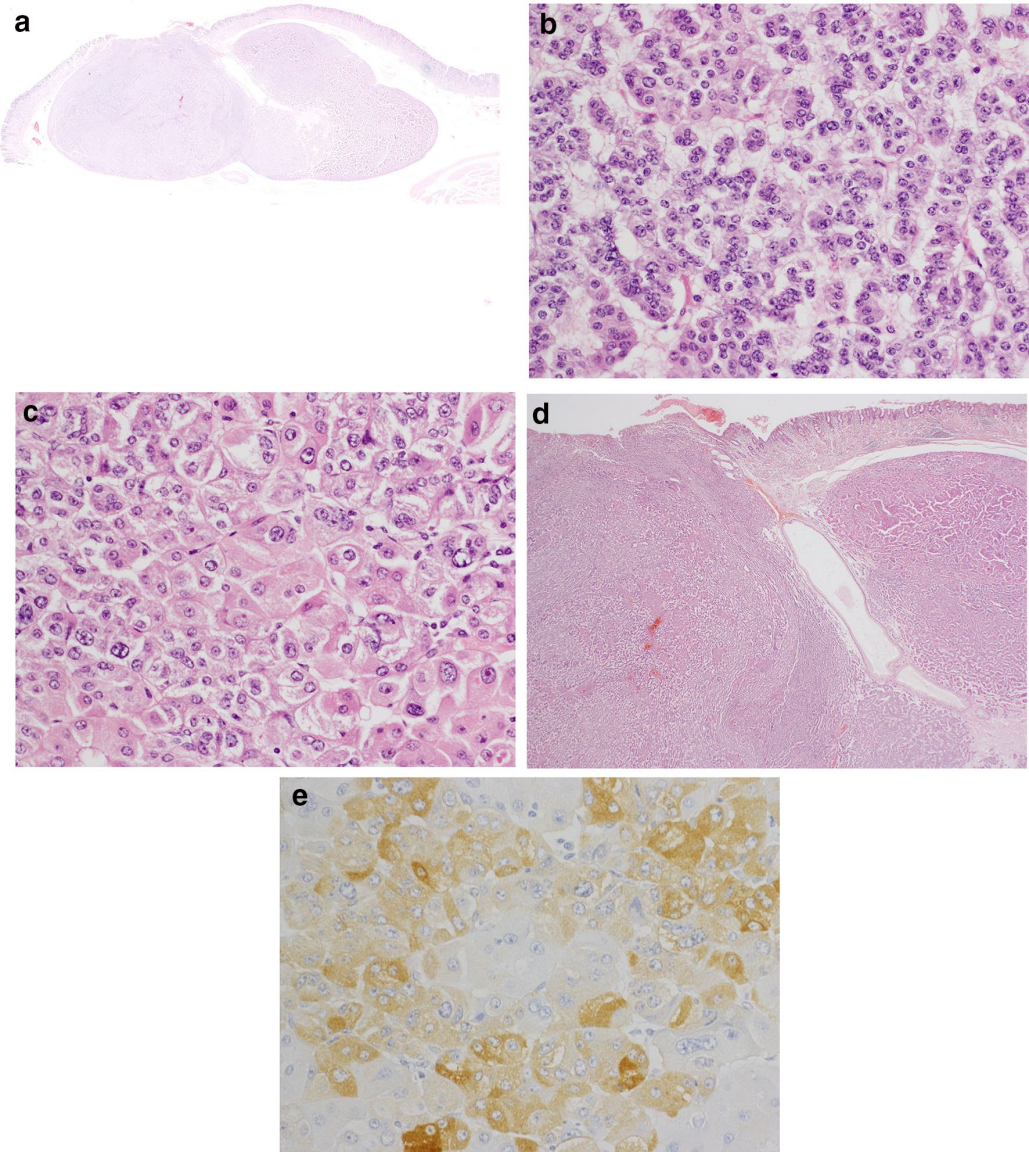
Histological findings. The mucosal aspect appeared slightly protuberant. An intramural, nodular, solid measuring 16 × 12 × 6 mm was noted. The tumor growth had a solid, nested, trabecular, and pseudoglandular pattern with delicate capillaries. **a** Aberrant exocrine acini were barely detected in the peripheral zone of the main tumor. **b** Large duct penetrating the center of the tumor. **c** Peripheral acinar cells were positive for trypsin

A 72-year-old woman underwent esophagogastroduodenoscopy for the first time in her life, as part of a general health check-up. An endoscopic examination revealed a 16-mm submucosal tumor on the greater curvature of the gastric body on a background of chronic atrophic gastritis due to *Helicobacter pylori* infection. The tumor had a smooth surface with a central depression and a small erosion on top (Fig. 1a, b). An abdominal contrast-enhanced computed tomography (CT) scan demonstrated a 16-mm diameter low-attenuated tumor in the greater curvature of the stomach with slow enhancement (Fig. 2). A CT scan of the abdomen and pelvis did not reveal any evidence of metastatic lesions. Endoscopic ultrasonography was not performed. Histological evaluation of the biopsy specimen revealed proliferation of small uniform cuboidal cells. The nuclei were monomorphic with inconspicuous nucleoli and cytoplasm was abundant. Immunostaining revealed that the tumor cells were positive for chromogranin A, synaptophysin, and CD56. The Ki-67 labeling index was < 2%. Therefore, based on the diagnosis of Grade 1 NET, laparoscopic and endoscopic cooperative surgery was performed. On gross examination, the mucosal aspect appeared slightly protuberant. An intramural, nodular solid mass measuring 16 × 12 × 6 mm was noted. Histological examination of the resected specimen revealed a cellular tumor primarily situated in the submucosa. The overlying mucosa was largely intact (Fig. 3a), except for a focal area where it was breached by the tumor. The mucosal surface showed focal ulceration. The tumor growth had a solid, nested, trabecular, and pseudoglandular pattern with delicate capillaries (Fig. 3b). There was minimal cellular pleomorphism. Mitotic counts were < 1 for each 10× high-power field. Tumor necrosis was not present. Aberrant exocrine acini were barely detected in the peripheral zone of the main tumor and in a duct penetrating the center of the tumor (Fig. 3c, d). Peripheral acinar cells were positive for trypsin (Fig. 3e). Although there were no islands of Langerhans, large duct and acini indicated the presence of ectopic pancreatic tissue. Ductular cells were negative for both neuroendocrine marker and trypsin. Immunohistochemical examination revealed that the tumor cells were positive for the neuroendocrine markers chromogranin A and synaptophysin and negative for gastrin, glucagon, somatostatin, VIP, insulin, and serotonin. The Ki-67 labeling index was 0.61%. These morphological features were typical of a well-differentiated Grade 1 NET. Adjacent gastric epithelium did not show evidence of endocrine cell hyperplasia.

## Discussion

The usual location of heterotopic pancreas is the stomach in 25–38% of cases, the duodenum in 17–35% of cases, and the jejunum in 15–21.7% of cases. It is rarely found in the esophagus and gallbladder. Gastric lesions are discovered in the antrum in 85–95% cases either on the posterior or anterior wall, being more common along the greater curvature [10]. Many gastric NETs arise in the body or fundic mucosa; the location of the tumor in our case cannot rule out heterotopic pancreatic origin. Guillou et al. [11] suggested three criteria for the diagnosis of adenocarcinoma arising within the setting of heterotopic pancreas. First, they stated that the tumor must be within or near the heterotopic pancreas; second, a transition between the pancreatic tissue and the tumor should be established; and third, the non-neoplastic pancreatic tissue should show well-developed ducts and acini. As the lesion in the present case was a NET, it was difficult to meet the second criterion. However, the diagnosis of NET arising from heterotopic pancreas was reasonable because the findings were compatible with the other two criteria.

A malignancy arising from heterotopic pancreas is exceedingly rare [6–8]; however, several reports have been published. Chetty and Weinred [9] reported the only other case of NET occurring from ectopic pancreas. Their case was an 85-year old man who had a Grade 1 NET, with a small Grade 3 focus. In our case, the NET was purely composed of a Grade 1 component. We could not refer to their procedures because the prognosis of their patient was not described. We presumed they had the same clinical outcome because histologically it was exactly the same as pancreatic origin and, to date, there is no evidence that they are clinically different. Close follow-up is essential and further research is needed to clarify the nature of NETs arising from gastric heterotopic pancreas. In practice, pathologists and endoscopists should be aware of the occurrence and association of NETs with pancreatic heterotopia in the stomach.
